# Old Atlanta Medical College

**Published:** 1883-10

**Authors:** 


### OLD ATLANTA MEDICAL COLLEGE.

This highly successful and venerable institution has just opened its twenty-seventh winter course with a larger class than has appeared there since the recent war. Not only is the present class superior in numbers, but there is an appearance of tone and intelligence about its members that gives promise of success and honorable usefulness in the future.

The Atlanta Medical College is among the foremost institutions of the kind in the South, and in the thoroughness and extent of its scientific teaching is scarcely surpassed by any school at the North.

